# “It pushes me, but it also drains me”: A qualitative study of perfectionism and well-being among doctoral students in India

**DOI:** 10.1080/17482631.2026.2760664

**Published:** 2026-11-11

**Authors:** Priyanka Sugumar, Samyukta Vaidyanathan

**Affiliations:** a Department of Clinical Psychology, Manipal College of Health Professions, Manipal Academy of Higher Education, Manipal, India

**Keywords:** Perfectionism, doctoral students, doctoral well-being, higher education, India, qualitative research

## Abstract

**Purpose:**

Although perfectionism has been linked to academic achievement and psychological functioning, less is known about how doctoral students experience, interpret, and negotiate perfectionism in their academic lives, particularly within Indian higher education. This qualitative exploratory study examined how doctoral students in India experience perfectionism.

**Method:**

The study was conducted between October 2024 and March 2026 following institutional ethics approval (Ref: IEC2: 177/2025). Thirteen doctoral students were recruited purposively after screening for sociodemographic variation and scores on the Frost Multidimensional Perfectionism Scale. Data were collected through in-depth semi-structured interviews and analyzed using thematic analysis.

**Results:**

Five themes were identified: Making sense of perfectionism, Expressions of perfectionistic tendencies, Influences on perfectionism, The double-edged experience, and Managing perfectionism. Findings suggest that perfectionism was experienced as both rewarding and exhausting, dynamically shaped by personal standards, academic cultures, supervisory relationships, familial expectations, and broader socio-cultural ideals of achievement.

**Conclusions:**

By situating perfectionism within the Indian doctoral context, this study contributes to the literature on mental health and well-being in higher education. Findings may inform doctoral supervision, institutional support systems, and interventions aimed at addressing maladaptive perfectionism while supporting students’ well-being.

## Introduction

Doctoral studies mark a crucial developmental phase in an academic career, requiring students to move from structured roles toward independent research identities, while navigating prolonged evaluation, uncertainty, and institutional expectations (Blomberg, 2025; Schmidt & Hansson, 2018). Paralleling the growth in doctoral education in East Asia broadly (Shin et al., 2018), India has also noted a steady increase in individuals pursuing doctoral studies. According to the Ministry of Education, doctoral students represent 2.7% of university enrolments, and the compound growth rate for enrolments to the programme was recorded as 11.1% over a period of five years since 2019 (All India Survey of Higher Education, 2024).

Doctoral students form a distinct subgroup within the general body of students, especially in view of the rigorous demands associated with the programme. They have been acknowledged as confronting distinct stresses and vulnerabilities, including heightened academic pressures, uncertainty regarding future career prospects, social isolation, resource constraints, and inadequate support networks, particularly in overburdened yet rapidly expanding higher education systems such as India (Berry et al., 2020; Guo & Liu, 2026b; Mahsood et al., 2025; Parveen et al., 2025). Recent research on the mental health of doctoral students further reveals consistently elevated levels of psychological distress, along with symptoms of anxiety and depression, and suicide risk (Satinsky et al., 2021). Longitudinal data also indicate a similar pattern of crisis, as evidenced by trends of increased rates of psychiatric medication use among doctoral students over the course of their study (Bergvall et al., 2025), adding to concerns about their well-being.

In light of such concerns, it would be important to understand factors that shape how they respond to challenges that accompany their academic journey. Perfectionism, which has been associated with stress and well-being in different contexts (Fernández et al., 2022; Filipkowski et al., 2021), may be especially likely to be activated by the doctoral training context, where students produce original work under conditions of uncertainty, delayed feedback, high self-direction, and repeated evaluation. It hence represents a relevant personality attribute for understanding their academic experiences.

Early psychodynamic accounts describe perfectionism as a defensive mechanism employed to protect against threatening feelings of worthlessness and shame arising from unrealistic and demanding parenting standards, thus theorizing it as inherently maladaptive and potentially draining of energy and resources (Horney, 1951). Later theorists, however, distinguished between normal and neurotic perfectionists, acknowledging that perfectionism does not itself constitute pathology (Hamachek, 1978). This distinction between adaptive and maladaptive dimensions has shaped subsequent conceptualizations (Slaney et al., 2001).

Other theories address the multidimensional nature of perfectionism, incorporating intrapersonal and interpersonal elements. Hewitt and Flett (1991), in this regard, described self-oriented, other-oriented, and socially prescribed perfectionism as personality styles with cognitive and motivational components (Hewitt & Flett, 1991). Frost et al. (1990) on the other hand, considered perfectionism as involving a drive towards reaching high standards and an overly critical nature of self-evaluations, with parental influences shaping the development of such tendencies (Frost et al., 1990), thereby conceptualizing perfectionism in terms of both personal and relational components while integrating adaptive and maladaptive facets.

Qualitative studies on perfectionism have considered how individuals perceive its influence on their performance and well-being (Binder et al., 2023; McKay et al., 2024; Tan, 2022), with some investigations identifying perfectionism as an important factor in shaping doctoral experiences (Jackman et al., 2022; Liardét, 2024; Mahsood et al., 2025). However, research specifically examining perfectionism among doctoral students remains limited. Further, although personal standards scores were comparable between doctoral and undergraduate students, the former scored higher on discrepancy, reflecting gaps between desired and actual performance (Moate et al., 2019). These differences support the need to examine perfectionism within everyday doctoral life.

Moreover, existing research on doctoral students has largely emerged from Western and high-income contexts, leaving limited understanding of how perfectionism is experienced in sociocultural and institutional settings such as India, where perfectionistic expectations may be negotiated through academic demands, family expectations, social comparison, competitive labour markets, and hierarchical supervisory relationships.

While quantitative studies identify associations between perfectionism and psychological outcomes, they may not fully capture how doctoral students interpret and navigate these tendencies. Accordingly, the present qualitative study aimed to explore how doctoral students in India experience and make meaning of perfectionism. Specifically, it sought to understand how perfectionism is conceptualized, how it manifests in doctoral life, what factors shape it, and how students navigate its perceived benefits and challenges.

## Methods

### Study design and setting

A qualitative exploratory study design was employed, involving data collection through in-depth semi-structured interviews and subsequent thematic analysis of the obtained data. The study was conducted at the Department of Clinical Psychology at Manipal College of Health Professions, Manipal, with the overall study period extending from October 2024 to March 2026, including approvals, recruitment, data collection, transcription, analysis, and write-up. Interviews were conducted between August 2025 and January 2026 via a secure online platform, namely Microsoft Teams, as per mutual convenience of participants and the researcher, and audio-recorded. No one other than the interviewer was present at the setting during the conduction of interviews.

### Participants and sampling procedure

Participants in the study comprised doctoral students aged between 24 and 36 years who were enrolled in full-time doctoral programmes in India and had completed at least one year of their doctoral studies. Eligibility additionally required a total score of ≥58 on the Frost Multidimensional Perfectionism Scale (FMPS). The FMPS was administered in its original English-language version, consistent with its use in previous research involving Indian samples, while participation was restricted to English-speaking doctoral students. A total score of ≥58 across items was used as a researcher-selected eligibility threshold, agreed upon by the principal investigator and supervisor for purposive recruitment. It was intended as a broad screening criterion for the qualitative exploration of perfectionistic tendencies, rather than a validated clinical cutoff or a basis for classifying participants as having high perfectionistic tendencies. While this screening criterion considered findings from recent empirical studies on the mean scores obtained by comparable samples of students (Ahmed et al., 2021; Pannhausen et al., 2022) as a yardstick, no formal calculation procedure was used to establish this specific threshold. Additional inclusion criteria required participants to be English-speaking and have access to devices and internet necessary to participate in the online interview. On the other hand, candidates were excluded if they were working jobs beyond their doctoral programme or had a history of diagnosis or treatment of a psychiatric illness, to maintain the focus of the study on perfectionism within the doctoral context.

Purposive sampling was employed to recruit participants with relevant experiences who could provide in-depth, informed responses about their own perfectionistic tendencies. Flyers were shared through academic institutions and the professional online platform LinkedIn, requesting interested candidates to reach out to the researcher. Of the 50 candidates who expressed interest in participating in the study, 22 candidates were found to be eligible to participate. The final sample comprised 13 participants. Of the remaining nine eligible candidates, five could not be contacted, and four reported insufficient time to participate. Thus, all eligible candidates who could be contacted and were available to participate were interviewed. During the final two interviews, the principal investigator informally noted repetition of previously described experiences, with limited additional information relevant to the study aim. Following the thirteenth interview, the dataset was considered sufficiently rich to address the study aim. Formal coding and theme development occurred subsequently; therefore, no formal assessment of code, thematic, or meaning saturation was undertaken.

As the study aimed to develop an in-depth qualitative understanding rather than draw statistical conclusions, no statistical sample-size formula or power calculation was used. Sample adequacy was considered retrospectively using the concept of information power (Malterud et al., 2016). Participants' direct experience of perfectionism within doctoral study and the detailed accounts generated through in-depth interviews supported the suitability of the dataset for addressing the aim of the study. Sociodemographic characteristics were collected and described to provide context for interpreting the findings.

### Researcher characteristics, reflexivity, and credibility

Data collection and analysis were primarily undertaken by the principal investigator and first author, a female postgraduate trainee in clinical psychology with training and certification in qualitative research methods and data analysis. The interview schedule was developed jointly with the research supervisor and corresponding author, a clinical psychologist. The principal investigator conducted the interviews, transcribed the recordings, and undertook initial coding. Codes, interpretations, themes, and subthemes were subsequently discussed with and reviewed by the supervisor, including consideration of alternative interpretations. An additional co- supervisor also reviewed the research notes. No prior relationship existed between the principal investigator and participants. Participants were informed about the role of the researcher and the purpose of the study, and rapport was established at the beginning of each interview. The schedule invited participants to describe their own understanding of perfectionism and reflect on its relevance to their doctoral experiences, including reference to their earlier FMPS responses. Qualifying phrases such as “if any” and “if at all” allowed participants to describe connections or an absence of connections.

The clinical psychology backgrounds of the principal investigator and supervisor informed an initial understanding of perfectionism as neither inherently beneficial nor harmful, with manifestations and degrees varying across individuals. While this perspective encouraged attention to individual differences, it may also have sensitized the researchers to particular interpretations of participants' experiences. As reflective field notes were recorded immediately after each interview, these notes and supervisory discussions were used to examine the researchers' assumptions and revisit interpretations in relation to accounts of participants.

Transcripts and interview summaries were shared with participants for review, and five participants provided feedback. This feedback was considered as an additional perspective during interpretation and informed refinement of theme labels and organization of subthemes. Supervisory discussions supported reflection on developing interpretations and their grounding in participants' accounts. An audit trail documented analytical decisions, including revisions to codes and themes and reflections on dataset adequacy.

### Data collection procedure

After the research proposal was approved by the Departmental Committee, approvals were sought from the Institutional Research Committee and the Institutional Ethics Committee. The study was then registered with the Clinical Trials Registry of India. Following the obtaining of due approvals, purposive sampling was employed to recruit participants. Sociodemographic information and responses to the FMPS were collected through an online form from willing candidates who expressed interest in participating in the study. Eligible participants were contacted for the scheduling of interview appointments, which were fixed as per mutual convenience of the participants and the researcher. Details about the study were explained through the provided participant information sheet, and informed written and verbal consent for participation and recording of interviews were obtained prior to the commencement of each interview. Rapport was established, and the interview was guided by a semi-structured schedule that involved nine open-ended questions along with a set of additional questions to probe into the experiences of participants.

Prior to primary data collection, the semi-structured interview schedule developed by the researchers for use in the current study was validated by seven experts in psychology, academia, and qualitative research, and pilot interviews were conducted with a small sample of three individuals closely paralleling the target population to further refine the schedule.

Following the conclusion of the interviews, which were conducted in English and lasted between 50 and 70 min each, audio recordings were transcribed by the researcher and analyzed as per procedure. No repeat interviews were conducted.

### Data analysis and coding

Data were analyzed using primarily inductive reflexive thematic analysis, guided by Braun and Clarke's six-phase approach and subsequent methodological refinements (Braun & Clarke, 2006, 2019). Situated within a constructivist perspective, the analysis explored how doctoral students experienced and made-meaning of perfectionism. The principal investigator actively developed codes and themes through engagement with participants' accounts, while recognizing that the research questions and interview schedule shaped the topics explored. The process was recursive, involving movement between transcripts, coded extracts, and developing themes. Transcripts were checked against audio recordings and read repeatedly for familiarization. Initial coding involved systematic line-by-line engagement with the transcripts, attending to the context and meaning of statements relevant to the research questions. Coding was conducted manually, with Microsoft Excel used to organize codes, supporting extracts, and developing thematic structure. Related codes were grouped to develop candidate themes and subthemes. These were reviewed against the coded extracts and the complete dataset, with their boundaries, distinctiveness, and central meanings refined before final definition and naming. Selected quotations were then incorporated into an analytical narrative addressing the research questions. The principal investigator led coding and theme development. The research supervisor reviewed the developing codes and themes, with discussions used to question assumptions, examine alternative interpretations, and enhance analytical depth. These discussions supported reflexive interpretation rather than establishing coding reliability or a single objectively correct interpretation. Reflective notes and an audit trail documented analytical decisions and revisions throughout the process. While COREQ (Tong et al., 2007) informed the initial reporting, during revision, reporting was reconsidered in light of Braun and Clarke (2025) values-based guidance, with attention to methodological congruence, theoretical positioning, reflexivity, and transparency in describing the analytical process (Braun & Clarke, 2025).

### Ethical considerations

Permission was obtained from the authors of the screening tool used, namely the FMPS (Frost et al., 1990). The study was initiated only upon approval from the Institutional Ethics Committee (Ref: IEC2: 177/2025), and data collection proceeded after registering the study with the Clinical Trials Registry of India (Registration Number: CTRI/2025/06/088489).

In preserving the rights of participants, the researcher ensured that they retained their choice in being able to withdraw from the study at any stage. Confidentiality was maintained and de-identification was established by assigning of alphanumeric codes to participants, in preserving their privacy throughout the process. Interviews were conducted through secure online platforms, with informed written and verbal consent preceding conduction and recording of interviews. Audio recordings of interviews were handled in password-protected systems, with plans for preservation for three years after the completion of the study. The study protocol included appropriate referral information for trusted mental health professionals made available in case of any distress experienced during the study process, though no such instance was reported by participants.

## Findings

Sociodemographic characteristics of the 13 participants are presented in Table I. Participants had a mean age of 28.5 years and 11 of them were women. While ten participants resided in urban areas, three reported living in rural settings. Geographically, five of them resided in Tamil Nadu, four in Karnataka, two in Kerala, and one each in West Bengal and Gujarat.

**Table I.** Sociodemographic characteristics of participants (*n* = 13).

Participant code	Age	Gender	State of residence	Residence setting	PhD study discipline	Nature of institution	Time completed in PhD programme	Score on FMPS
P1	30	Female	West Bengal	Urban	Chemistry	Government	6 years	75
P2	30	Female	Tamil Nadu	Urban	Microbiology	Private	6 years	93
P3	34	Female	Karnataka	Urban	Adolescent Mental Health	Private	1 year 11 months	101
P4	28	Female	Karnataka	Urban	Audiology	Private	3 years	92
P5	33	Female	Tamil Nadu	Urban	Journalism and Communication	Government	4 years	110
P6	25	Female	Gujarat	Rural	Organizational Behaviour and HR	Government	2 years	90
P7	30	Female	Tamil Nadu	Urban	Management	Government	2 years	99
P8	25	Female	Karnataka	Rural	Organizational Behaviour	Government	1 year	85
P9	31	Male	Tamil Nadu	Urban	Aerospace	Government	3 years	85
P10	27	Female	Kerala	Urban	English	Govt- Aided	3 years	60
P11	25	Male	Karnataka	Rural	Chemistry	Private	1 year 7 months	71
P12	27	Female	Kerala	Urban	English	Government	3 years	106
P13	26	Female	Tamil Nadu	Urban	Psychology	Govt- Aided	1 year	97

*Note*: *P = Participant.

**Table II.** Themes and corresponding subthemes.

Number	Theme	Subthemes
1.	Making sense of perfectionism	1.1 Personal meanings 1.2 Identifying as a perfectionist 1.3 Evolving standards 1.4 Justifying perfectionism 1.5 Misconceptions encountered 1.6 Unspoken realities
2.	Expressions of perfectionistic tendencies	2.1 Self-imposed standards 2.2 Striving for perfection 2.3 Responses to outcomes 2.4 High standards in collaborative work
3.	Influences on perfectionism	3.1 Individual factors 3.2 Family influences 3.3 Sociocultural expectations 3.4 Supervisory influences 3.5 Institutional standards
4.	The double-edged experience	4.1 Perceived advantages of strivings 4.2 Performance-related costs and academic strain 4.3 Impacts on health and well-being
5.	Managing perfectionism	5.1 Personal coping strategies 5.2 Use of support systems 5.3 Unmet needs and recommendations

In terms of social background, 11 participants identified as upper-middle class, 12 lived in nuclear families, and 11 were single or unmarried. Academically, seven attended government institutions, four attended private institutions, and two were part of government-aided institutions. Participants represented diverse academic disciplines, with three each from Science, Health Sciences, Humanities, and Management, and one from Engineering. Participants had completed between one and six years of their doctoral programmes, and eight were first-generation doctoral students. All participants scored at or above the FMPS screening threshold of 58.

Codes generated from data analysis were organized into 21 subthemes and 5 broader themes: (1) making sense of perfectionism, (2) expressions of perfectionistic tendencies, (3) influences on perfectionism, (4) the double-edged experience, and (5) managing perfectionism (Table II).

### Theme 1: Making sense of perfectionism

This theme captures how participants understood perfectionism and situated themselves in relation to their perfectionistic tendencies. It explores the subjective frameworks through which doctoral students interpreted these tendencies in themselves, through six distinct subthemes: (1) personal meanings, (2) identifying as a perfectionist, (3) evolving standards, (4) justifying perfectionism, (5) misconceptions encountered, and (6) unspoken realities.

“*I think it has taken 2 years for me to finally, actually even understand that oh I have been a little perfectionist… So my whole understanding or acceptance that oh, maybe I am kind of in that cycle, itself, I think, was a journey all this while.” (P7)*

Within sub-theme *1.1 Personal meanings*, participants offered subjective definitions of the term, often specifying that these were individual interpretations beyond standard academic definitions. While their action-oriented descriptions concerned being thorough and “acing” tasks, outcome-oriented descriptions involved defining the trait as producing the “best possible version” of their work or meeting specific expectations concerning presentations and publications. These accounts located perfectionism in both the process of working and the standards by which completed work was judged. Subtheme *1.2 Identifying as a perfectionist* further captures variation in how participants related these meanings to themselves. In this regard, while many participants explicitly applied the label to themselves, some others expressed ambivalence in accepting the identity of a “perfectionist”, often citing areas of life where strivings were not consistently present. Identification therefore involved considering both the presence of these tendencies and how fully the label described the person.

*“I know that I do have few elements of perfectionism… yeah, it is not surprising to me. I do know I am a perfectionist.” (P6)*

Under subtheme *1.3 Evolving standards,* participants described changes involving their perfectionistic tendencies during their doctoral studies, indicating that these tendencies were dynamic rather than fixed. For some, experiences in which effort did not produce the expected outcomes prompted greater flexibility in setting standards and evaluating performance.

*“Whatever you decide on doesn't really happen that way… Like you set goals and it does happen that way, it never is like that. So based on all of these reasons, my very stringent criteria of effort to reward changed. It became more flexible.” (P4)*

However, adjustment was not uniformly experienced as increased flexibility. While one participant described a profound crisis as established plans became less dependable, others reported intensified perfectionistic strivings. These contrasting accounts suggest that doctoral experiences could unsettle existing standards without necessarily reducing the pressure to achieve.

Even with such differences, within subtheme *1.4 Justifying perfectionism*, participants described the perceived value of such tendencies and their continued relevance. These justifications included satisfaction with performance outcomes and a sense that striving for perfection was the “right” approach. For some, perfectionism consequently carried personal or moral significance beyond its practical benefits. An explicit unwillingness to change these tendencies illustrated their continued value to participants, even alongside accounts of strain.

*“I don't think I might change anything because that is what gives me satisfaction.” (P2)*

Building on such reflections on their perfectionistic tendencies, within subtheme *1.5 Misconceptions encountered,* participants resisted explanations that they perceived to have misrepresented their own experiences. Assumptions that perfectionism is always internally driven or rooted in superiority were challenged, as it may alternatively be externally reinforced and connected to feelings of inadequacy. Their accounts thus highlighted a difference between how perfectionism might appear to others and the concerns underlying it.

*“From outsider perspective, perfectionists, people may think that we are striving for superiority… but in reality, it could be stemming from that sense of inferiority.” (P6)*

Relatedly, within subtheme *1.6 Unspoken realities,* participants described aspects of perfectionism that remained less visible, noting that high-quality outputs could attract appreciation while concealing the effort, strain, and health costs involved in producing them. Participants also described the difficulty of recognizing or accepting their own perfectionistic tendencies, suggesting that these experiences could be unacknowledged by both others and themselves.

*“I think people are happy when they get the results… but they don't see the hardship that we go through behind getting those so-called good papers.” (P11)*

Taken together, these subthemes illustrate the ways in which participants construct meaning around perfectionism, from personal definitions and resonance with the identity to reflections on its role, misconceptions, and unspoken dimensions. These suggest that perfectionism was not experienced simply as a stable personality trait, but as a negotiated and evolving orientation.

### Theme 2: Expressions of perfectionistic tendencies

This theme captures how perfectionism manifested in one's expectations for oneself and the drive to achieve these expectations, with reference to doctoral studies. It records the varied ways in which doctoral students expressed their perfectionistic orientations, through four distinct subthemes: (1) self-imposed standards, (2) striving for perfection, (3) responses to outcomes, and (4) high standards in collaborative work.

*“I have a timeline for myself… I know I put this on myself, but then, for me, that is my expectation.” (P13)*

Subtheme *2.1 Self-imposed standards* considers the criteria set by participants for themselves during the doctoral journey, often exceeding institutional requirements. Such criteria included completing work before formal deadlines and other specific performance standards for coursework, data collection, writing, and publications, with an emphasis on the quality of outcomes. These standards tended to be associated with concerns of a lack of satisfaction or negative self-evaluation, highlighting the underlying perfectionistic strivings.

*“I would say the research paper that I generate, that I write, it should be of a particular quality. I mean, it should be well written, it should have a solid argument. The archives should be perfect. I mean, it should be well composed, all of that… or even start writing the draft of my thesis. That too a good draft, nothing average. I don't think I'll be satisfied with an average draft.” (P12)*

Within subtheme *2.2 Striving for perfection*, participants described how they sought to reach these set standards, through meticulous planning and repeated efforts to refine their work. Their approaches ranged from using checklists and preparing for contingencies to constantly reviewing and reworking drafts or striving to deliver good output in the first attempt. These practices reflected different ways of managing the demands of high standards, while the emphasis remained on ensuring that the final output met personal expectations.

*“I tend to work a lot on something, even if it takes a lot of time. I spend that time to make sure that I get satisfied, like I am satisfied with the result.” (P2)*

Further, under subtheme *2.3 Responses to outcomes,* doctoral students recounted how they responded when these efforts led to either success or perceived shortcomings. Achieving their standards tended to bring happiness and confidence, although such satisfaction was sometimes short-lived as attention quickly shifted to the next task. Conversely, unmet expectations were reported to result in frustration and disappointment. Further, participants also noted that their evaluation of a “perfect” output often changes over time, leading them to later regret work they previously found satisfactory, highlighting how achievement could bring satisfaction without necessarily providing lasting reassurance about the quality of one's work.

*“I'm not very elated when I complete things, because at the back of my mind, there's always an, okay, what's the next thing to do? But I just feel relieved that one thing is ticked off.” (P3)*

Under subtheme *2.4 High standards in collaborative work,* participants further noted that perfectionistic tendencies extended beyond individual work, influencing expectations within group contexts. These included taking responsibility for important tasks or over-compensating by doing others' work when they perceive it as falling short of their high standards, often despite experiencing reluctance.

*“I think it [perfectionism] shows up in the sense that, maybe I will take more initiative or maybe I will, you know, take up the harder task.” (P6)*

These subthemes collectively illustrate the multifold manifestations of perfectionism across participants' thoughts, behaviours, emotions, and interactions with others, beyond simple characterization by high standards. Participants' accounts demonstrated a cyclic pattern in this regard, whereby perfectionism drove them to make meticulous efforts to meet their own rigorous expectations, resulting in only temporary satisfaction before subsequent tasks reactivated the same high standards and frustration when these expectations remained unmet.

### Theme 3: Influences on perfectionism

This theme captures the intrapersonal and contextual influences shaping perfectionistic tendencies. It highlights the various internal and external contributors to perfectionistic standards and strivings, across five distinct subthemes: (1) individual factors, (2) family influences, (3) sociocultural expectations, (4) supervisory influences, and (5) institutional standards.

Under subtheme *3.1 Individual factors* most participants identified intrapersonal drivers as key influences on their perfectionistic strivings. In this regard, they noted an inherent need for control and constant internal pressure to be flawless as prompting perfectionism, alongside tendencies to compare and compete with high-achieving peers. Additionally, fear-based motivations, such as concerns regarding rejection and negative evaluation by others, were also described as shaping their perfectionistic tendencies.

*“I think it is my basic nature, that also comes into academics, doing the best, best for me, best for others.” (P10)*

Beyond such intrinsic characteristics, subtheme *3.2 Family influences* captured varied ways in which family experiences shaped participants' perfectionistic strivings. While some participants perceived no parental influence, others reported internalizing parental standards and rules, comparing themselves with high-achieving family members, or seeking parental approval. Interestingly, one participant also described feeling motivated by a lack of explicit expectations from their family, viewing it as an unwritten mandate to achieve.

*“For his [father’s] ‘society’ concerns as well, he expects me to be at certain level, so he will expect me to be perfect… when my parents, like obviously set some rules that you have to behave in so and so and so ways, then when you grow up and think what that meant, it was true, it was good for me… now it is personal perfectionism” (P11)*

Further, within subtheme *3.3 Sociocultural expectations,* some participants noted that their perfectionistic strivings were also motivated by a drive to counter conservative norms or prove themselves in the face of gender-based underestimation.

*“Again, cultural expectations. When I turned around 22, 23, there was a lot of, you know, are you not getting married? You know, these proposals. I said, no, I need to study. I need to do something… So somewhere now, because I’m unmarried, I think, subconsciously, there might be a need for me to keep proving myself.” (P3)*

Within the academic context, subtheme *3.4 Supervisory influences* captures how supervisors shaped doctoral students' perfectionistic strivings through explicit expectations and high standards. Participants also described how these expectations could become internalized as personal standards, while positive feedback and appreciation sometimes reinforced their efforts to meet these standards, highlighting how supervisory influence could operate beyond direct expectations.

*“They [supervisors] want good papers, they want good results. They want the best PhD student, they want the best research scholar that is possible. So at the end of the day, it’s kind of the supervisor that comes up, and that gets ingrained in you.” (P1)*

Such influences of guides and supervisors were situated within a broader institutional context that shaped their expectations and performance in subtheme *3.5 Institutional standards*. Tacit norms for high achievement and an implicit competitive culture tended to be major factors, with specific institutional criteria for degree eligibility, such as publication requirements, also shaping the personal standards of doctoral students.

*“nowadays it is like publish or perish... the race is getting tighter and tighter because there are not much employment opportunities, as I told you before, which means you have to build an excellent academic profile, which means that your thesis should be good. Not good, actually more than good. We should have excellent publications and presentations” (P12)*

Overall, this theme demonstrates that perfectionism tended to be experienced as a product of interacting influences across personal, relational, cultural, and contextual levels.

### Theme 4: The double-edged experience

This theme captures the paradoxical nature of perfectionism by considering perceived benefits of such tendencies as well as the associated costs. It highlights the contradictory influences of perfectionistic orientations across three subthemes: (1) perceived advantages of strivings, (2) performance-related costs and academic strain, and (3) impacts on health and well-being.

As captured by subtheme *4.1 Perceived advantages of strivings*, most participants reported benefits associated with perfectionistic tendencies during the doctoral journey, especially regarding performance. Perfectionism played a role in helping persist in tasks and produce high-quality work, while some also credited it for facilitating efficiency in meeting deadlines while maintaining quality. Further, specific payoffs in doctoral tasks, such as obtaining optimal grades in coursework and achieving manuscript acceptance for publication, were also highlighted as advantages conferred by perfectionistic strivings.

*“I think that [perfectionism] helped me, you know, to be there till the end, survive. What do I say? Yeah. I should be there till the end and perform well also… Like okay, output is coming good.” (P6)*

Contrastingly, under subtheme *4.2 Performance-related costs and academic strain* several participants noted the accompanying negative consequences of perfectionism for achievement and research processes. In this regard, they described how repeated reviewing and fear of failure made it difficult to initiate or complete tasks, contributing to delays in doctoral progress, while rigid expectations for written work were associated with writing blocks.

*“I think with respect to my perfectionism, how it has hindered me also, not as a boon, but in work, would especially be the delay, the constant review.” (P4)*

*“I have not published yet. Because there is still a small fear of whether I would maintain a standard in what I submit.” (P5)*

Beyond productivity and performance, under subtheme *4.3 Impacts on health and well-being* participants also described the consequences of perfectionism for physical, psychological, and social health. Striving for perfection often led to neglecting essential health practices like adequate sleep and diet. Further, participants also highlighted psychological impacts, including burnout, frustration, and impairments to memory and concentration, alongside impacts on social well-being and work-life balance, as perfectionistic strivings and accompanying guilt led to undervaluing other aspects of life over producing “perfect” output and completing one's doctoral tasks.

*“You are so focused on delivering a top-notch work, you’re so focused on doing something perfectly, doing something flawless that you will not care about anything else. Your physical health, mental health, emotions, everything else takes a backseat.” (P3)*

Accounts captured by this theme illustrate the dual nature of perfectionism in conferring benefits to performance as well as considerable costs that extend beyond academic performance to affect broader well-being.

### Theme 5: Managing perfectionism

This theme captures how participants coped with challenges that come with perfectionism and their recommendations for further enhancement. It records the various internal methods and external resources utilized by doctoral students to address the difficulties inherent in their perfectionistic orientations, across three distinct subthemes: (1) personal coping strategies, (2) use of support systems, and (3) unmet needs and recommendations.

Within subtheme *5.1 Personal coping strategies,* participants described specific techniques and practices employed to manage challenges associated with perfectionism. Deliberate cognitive efforts, such as self-talk, and the use of external reminders, were utilized to shift perspectives, revisit motivations, and set necessary boundaries regarding overworking.

*“I've made it a habit to tell myself it's okay, relax, to a point where I have to keep reminders on my phone, saying that, take a break. I’ve to make conscious efforts.” (P3)*

Moreover, many participants also reported incorporating breaks involving engaging activities, ranging from household chores to hobbies like baking or collecting flowers. They further indicated selecting these activities strategically based on the specific function sought, whether they required grounding, a sense of completion, or simple distraction.

Further, under subtheme *5.2 Use of support systems*, participants described drawing on both personal networks and structured institutional aid to cope with the challenges of perfectionism. Empathetic and informational support from friends and peers was highlighted as important in such coping, with those in similar academic environments described as uniquely capable of understanding and normalizing their struggles.

*“Obviously, peers are the major support because they know the situation best.” (P9)*

Parents and family members were also noted as providing both emotional reassurance and tangible assistance, while the role of guides was emphasized in providing reassurance and practical academic advice. Additionally, some participants also reported seeking formal counselling or therapy.

*“My parents, I would say, a lot of moral support, and they also, at times come here and stay with me so that I can focus on my research and complete my work.” (P7)*

*“Sometimes even your guide understands that you are very frustrated. So even he gives you some good counsel. In my case it has happened.” (P1)*

Building on reflections about existing coping resources, under subtheme *5.3 Unmet needs and recommendations* participants recorded evaluations of current mechanisms and provided suggestions for preventing or reducing the harmful effects of perfectionism. While some expressed satisfaction with available support and the value of existing coping resources, several participants also emphasized the need for personal transformations, including fostering greater flexibility in evaluations and addressing avoidance-related procrastination, taking personal responsibility for changing themselves even when external influences contributed to the issues faced.

*“I don't think any external help can reduce this because I see this perfectionism more, more from an individual’s side... I don't think any external help would, you know, reduce this feeling, may be do something better, yeah.” (P8)*

At an institutional level, some suggested improved counselling facilities and faculty training, while others highlighted the critical role of guides in providing explicit direction to help them halt continuous, detrimental reviews of their work.

*“I think, the professors or guides, they must guide the student that, okay, this is a sufficiently good work, you can stop your work here and try for the next work. So I think their guidance will be crucial in dealing with challenges of perfectionism.” (P9)*

In all, accounts of coping mechanisms typically employed and recommendations made suggest that managing perfectionism may require a balanced integration of personal initiative, social support, and systemic institutional measures.

## Discussion

This qualitative exploratory study examined how doctoral students in India experience and make meaning of perfectionism within their doctoral journeys. The findings suggest that perfectionism was not experienced merely as a stable individual trait, but as a negotiated and contextually embedded orientation shaped by personal standards, academic expectations, supervisory relationships, familial and sociocultural influences, and institutional cultures of productivity. Participants described perfectionism as a double-edged experience, as it supported persistence, quality, and achievement, while also contributing to delay, writing blocks, emotional exhaustion, neglect of health, and compromised work-life balance. These findings align with prior propositions that perfectionism plays an important role in doctoral student well-being (Guo & Liu, 2026b; Mahsood et al., 2025), while further contextualizing this relationship within the lived realities of doctoral training in India.

### Perfectionism as a personally understood, negotiated identity

Participants' accounts suggest that perfectionism in the doctoral context may be viewed not as a fixed personality characteristic but as a rather idiosyncratic, dynamic process that shapes how students interpret, pursue, and evaluate their work. While the performance-oriented definitions offered by participants for perfectionism mirrored conceptualizations reported by Indian young adults (Sidharth et al., 2023), these accounts diverged from theoretical models that distinguish “excellencism” from perfectionism (Gaudreau & Benoît, 2025). This underscores the personalization recorded in these definitions, suggesting that perfectionism tends to be understood through subjective meanings. Consistent with these personal conceptualizations, participants' application of the label of a “perfectionist” to themselves aligned with findings from other student populations (McKay et al., 2024), while differing from broader Indian trends noting denial of such labels (Sidharth et al., 2023). This difference, although possibly interpreted as a tendency among doctoral students to identify with the label, may rather reflect the specific characteristics of the present sample, particularly the use of perfectionism-based screening.

Though participants' personal definitions tended to blur the distinction between perfectionism and excellencism as previously noted, their lived experiences more closely reflected theoretical descriptions of perfectionism. In this regard, descriptions concerned self-imposed high standards and rigid expectations across various domains of doctoral work, including coursework, data collection, and research performance. These align with the intense internal pressures documented among students in other demanding academic and professional contexts (Jasmin & Binnie, 2020; McKay et al., 2024), while contextualizing these expectations within the unique demands of doctoral training. Accounts of rigid benchmarks and the tendency to interpret failure in catastrophic terms also correspond to characteristic features of perfectionism (Gaudreau & Benoît, 2025). Participants' descriptions of these standards translating into meticulousness, persistent striving, and heightened attention to detail, as well as expressions of high standards within collaborative contexts, particularly through overcompensating for others' perceived shortcomings, similarly aligned with observations of perfectionistic strivings in broader academic and occupational settings (Ocampo et al., 2020; Tan, 2022). Further, cognitive and emotional responses linked to outcomes, including confidence contingent on performance and the absence of enduring gratification following achievement, paralleled existing descriptions of chronic inadequacy and restlessness (Binder et al., 2023; Starley, 2019), highlighting ways in which perfectionism manifests in the experiences of doctoral students. Taken together, these accounts suggest that perfectionism tended to operate as a cyclic pattern, whereby demanding self-imposed standards prompted persistent striving, with outcomes becoming crucial for evaluation of these efforts and achievements providing only temporary satisfaction before expectations were redirected towards subsequent academic demands.

Moreover, perfectionism and its cyclic pattern were described as dynamic as opposed to fixed, evolving in response to experiences in the doctoral context. In particular, a perceived mismatch between expectations and outcomes was reported, paralleling trends of higher discrepancy scores among doctoral students (Moate et al., 2019). However, while some students described developing greater flexibility over time as a response to such mismatch, others reported experiences of crisis associated with these tendencies. These differing trajectories may be shaped by a range of individual and contextual factors that were beyond the scope of the present study. Future research could investigate the potential roles of adaptability, resilience, mentorship styles, and institutional contexts in explaining these variations. Further, despite recognizing such evolving experiences and costs associated with the described cycle of strain, participants continued to defend their perfectionistic strivings, aligning with prior documentation of the trade-offs involved in perfectionism (Tan, 2022). This complexity could also explain the characterization of related struggles as “unspoken”, which may also reflect the influence of meritocratic academic cultures that overlook the difficulties underlying high performance (Binder et al., 2023; Tan, 2022).

### Perfectionism as personally driven and contextually produced

Findings also illustrate the various factors that contribute to and sustain perfectionistic standards and strivings, highlighting how perfectionism is shaped by multiple influences rather than stemming from a single source. Accounts of internal drives and fear-related motivations mirrored established theoretical models and empirical findings that associate perfectionism with both achievement-oriented and threat-related motivations (Binder et al., 2023; Filipkowski et al., 2021; Frost et al., 1990), with descriptions of competitive striving paralleling the association of maladaptive perfectionism with “useless superiority effort” (Çimşir & Ülker Tümlü, 2022).

These internal pressures, nonetheless, tended to be closely intertwined with external drivers. Descriptions of familial influences, particularly high parental expectations, as being significant were also consistent with broader findings across cultures (Ashraf et al., 2023; Tan, 2022). However, the comparatively limited emphasis on parental criticism differed from Western literature. This difference may reflect the way participants interpreted family expectations as guidance, responsibility, or support rather than criticism, although further research is needed to examine how parental expectations and criticism are understood and reported in Indian doctoral contexts. Adding to these cultural considerations, findings suggesting that the absence of explicit expectations could itself function as an implicit mandate to achieve represented a distinctive observation that may reflect culturally embedded understandings of family responsibility and achievement. Further, participants' descriptions of striving to challenge conservative gender roles and patriarchal norms indicated the influence of broader sociocultural expectations. This extends existing achievement-oriented literature, which tends to focus on competitive drives (Tan, 2022), highlighting how perfectionistic strivings may also serve as a means of negotiating identity and legitimacy within the Indian sociocultural context.

Within the doctoral environment, supervisory relationships and institutional norms were described as influencing the participants' perfectionistic tendencies. Consistent with previous findings (Jackman et al., 2022; Ocampo et al., 2020), high expectations of supervisors and their modelling of workaholism were reported as shaping these tendencies, while accounts of unintentional reinforcement through appreciative feedback offered a relatively underexplored perspective. Beyond such supervisory influences, meritocratic academic cultures were reported as playing important roles, consistent with prior literature (Gaudreau & Benoît, 2025), with an additional emphasis on specific institutional norms as frames of reference for one's own standards representing a more specialized contribution worthy of further exploration.

### The double edge of perfectionism in doctoral well-being

Participants' accounts of their experiences with perfectionism highlight a paradox, as the very perfectionistic tendencies that were perceived to facilitate academic achievement also appeared to have costs even beyond the professional domain, undermining health and well-being. Reports of producing high-quality output and meeting deadlines paralleled previous observations that perfectionistic strivings relate to higher productivity satisfaction (Gaudreau & Benoît, 2025) and academic achievement (Madigan, 2019). Further, descriptions of task-specific outcomes, such as optimal coursework grades and successful publication acceptance, extends existing literature by demonstrating how the perceived benefits of perfectionism are embedded within the distinctive demands and markers of success associated with doctoral training. Conversely, delays and repeated reviewing reported as stemming from fear of failure represent detrimental effects for performance, mirroring prior linking of perfectionism with academic procrastination (Agarwal & Joy, 2023) and the tendency to engage in excessive “tinkering” with language (Russell-Pinson & Harris, 2019). However, findings relating to writing blocks, described by participants as particularly hindering, appeared to differ from observations suggesting that perfectionists often maintained average productivity despite such challenges (Sala-Bubaré et al., 2018), indicating that perfectionistic tendencies may be experienced as more impeding in the doctoral context.

Moreover, participants reported impairments across physical, psychological, and social domains due to their perfectionistic strivings, which resonated with previous documentation of the adverse effects of perfectionism for multiple facets of well-being (Çimşir & Ülker Tümlü, 2022; Filipkowski et al., 2021), supporting the notion that achievement gains may come at considerable personal costs. Moreover, longitudinal evidence linking declining self-rated health with reduced life satisfaction during postgraduate education (Guo & Liu, 2026a) underscores the interconnectedness of health and broader well-being in academic settings. While such evidence does not directly examine perfectionism or doctoral students, it highlights the importance of attending to health along with academic progress. Considered alongside earlier findings demonstrating how perfectionistic strivings may be reinforced through supervisory relationships, publication expectations, and meritocratic institutional cultures (Gaudreau & Benoît, 2025; Jackman et al., 2022; Ocampo et al., 2020), these accounts suggest that doctoral environments may simultaneously reward behaviours associated with academic success while perpetuating practices that compromise students' well-being. Findings thus point to achievement and well-being as deeply intertwined outcomes of the same perfectionistic processes, underscoring the importance of doctoral environments that foster excellence without normalizing unsustainable standards of performance.

### Managing perfectionism: beyond individual coping

Participants further described the various strategies and resources used to deal with the challenges brought on by perfectionism, their evaluations of the mechanisms in place, and suggestions for improvements to enhance the way these challenges are managed. In this regard, accounts of the use of internal strategies such as self-talk and perspective-shifting for boundary-setting resonate with documented experiences of students in higher education (Jasmin & Binnie, 2020). Extending existing literature, participants described selecting self-care activities according to their intended functions, such as seeking grounding versus distraction, offering a more nuanced understanding of how these doctoral students tailored their coping approaches. The reported importance of support networks similarly mirrors the recognized role of social resources in doctoral well-being (Jackman et al., 2022). Further, research among postdoctoral researchers has also linked supervisory, financial, organizational, and career-development support to job satisfaction, with self-efficacy statistically mediating these associations (Liu et al., 2026). Although this cross-sectional study involved postdoctoral researchers rather than doctoral students, it highlights a potential connection between external support and personal confidence. Consistent with this, participants in the present study emphasized the value of reassurance and practical guidance, although the role of self-efficacy was not directly examined.

However, while participants prioritized peers within similar academic environments, differing from Western literature identifying romantic partners as primary sources of support (Evans et al., 2021), the reasons cited for the same, concerning shared academic contexts fostering empathy and normalization, were consistent with previous observations (Bogardus et al., 2021; Jackman et al., 2022). Participants also emphasized the value of family support and further portrayed supervisors as influential sources of reassurance whose understanding and realistic expectations could help mitigate perfectionistic pressures, differing from more functional, resource-oriented descriptions centred on productivity noted in Western literature (Evans et al., 2021), possibly reflective of the value placed on mentors and guides within participants' academic contexts.

Importantly, findings caution against locating responsibility for managing perfectionism solely within the individual. Participants' calls for broader systemic and “upstream” changes involving mentorship and institutional training, aligning with contemporary academic recommendations (Jackman et al., 2022), underscore this need, while the simultaneous emphasis on personal transformation add a further dimension to these accounts, possibly reflecting an internalized sense of responsibility.

Together, these findings highlight the interconnected role of individual, interpersonal, and institutional resources in shaping how perfectionism-related challenges are managed within the doctoral context, and point to important implications for enhancing the well-being of doctoral students.

The overall contribution of this study lies less in demonstrating the established benefits and costs of perfectionism than in examining how these were understood and negotiated within participants' doctoral lives. Accounts highlighted less explored nuances of how striving could acquire moral significance as the “right” approach, serve as a means of responding to gendered expectations, and be reinforced through supervisory appreciation and institutional productivity demands, alongside more readily identified advantages to academic performance. Participants also described differing trajectories involving their perfectionistic tendencies within the doctoral context, including greater flexibility, intensified striving, and subjective feelings of crisis. These contextual insights help explain why perfectionistic practices could remain personally valued despite their perceived costs. They are offered as situated interpretations of this sample's experiences, rather than patterns unique to India or representative of all doctoral students.

### Strengths, limitations, implications, and future recommendations

The findings of the study should be considered in light of certain limitations. First, the sample comprised 13 participants whose accounts supported an in-depth exploration of the study aim, as discussed previously through information power. However, participants were predominantly unmarried women from upper-middle-class backgrounds, limiting the range of perspectives represented and the transferability of findings to other doctoral populations. The study also included only full-time, English-speaking doctoral students, which may limit the transferability of findings. The researcher-selected FMPS eligibility threshold of ≥58 was not validated for identifying perfectionistic tendencies in this population and may have influenced the composition of the sample. Although participants did not uniformly attribute their experiences to perfectionism, prior completion of the FMPS and the explicit focus of the interview on the construct may have shaped the experiences they recalled and discussed. Further, psychiatric history was assessed through self-report rather than formal clinical screening. As candidates with a history of diagnosis or treatment for psychiatric illness were excluded, the study may not fully capture experiences of perfectionism among students with clinically significant psychological distress. Additionally, the use of online interviews may have influenced the nature and depth of disclosure, although it also allowed participants from different locations to take part. Moreover, the use of purposive recruitment and voluntary participation may have introduced self-selection bias, as students who chose to participate may have been more reflective about or affected by perfectionism. Despite these limitations, the study also has notable strengths. The qualitative exploratory design allowed the participants' own meanings, reflections, and lived experiences to guide the understanding of perfectionism, rather than relying on pre-existing quantitative assumptions. The inclusion of doctoral students from different academic disciplines and institutional types also provided a broader view of doctoral experiences within the Indian higher education context. Moreover, the use of a validated screening tool supported purposive recruitment of participants who endorsed perfectionistic tendencies, thereby enabling focused exploration of how perfectionism was experienced in relation to academic work, doctoral performance, well-being, and coping. Overall, the study contributes to an underexplored area by situating perfectionism within the lived realities of doctoral training in India.

Findings of the study have implications for doctoral supervision, institutional support, and student well-being initiatives. At the individual level, doctoral students may benefit from interventions that address rigid standards, self-critical evaluation, avoidance-related procrastination, and difficulty accepting work as “good enough”. However, as findings also suggest that contextual factors, including supervisory expectations, institutional norms, family expectations, and socio-cultural ideals of achievement, contribute to perfectionistic strivings, support efforts should include both individual and systemic components. These may include accessible counselling services, peer-support groups for doctoral students, supervisor senitization, clearer academic milestones, and institutional conversations around realistic productivity expectations and doctoral well-being.

Future research may include larger and more diverse samples to enhance the transferability of findings across doctoral contexts. Comparative studies involving full-time and part-time doctoral students, students across different disciplines, genders, socioeconomic backgrounds, and institutional types may further help clarify how contextual factors shape perfectionistic tendencies. Longitudinal studies may also be useful in understanding how perfectionism evolves across different stages of the doctoral journey. Quantitative and mixed-methods studies may further assess factors contributing to perfectionism among doctoral students and its impact on academic progress and well-being. Finally, intervention-focused research may explore the effectiveness of mentorship-based initiatives, peer support, counselling, and institutional well-being programmes in addressing the challenges associated with perfectionism among doctoral students.

## Conclusion

Experiences of perfectionism among doctoral students in India were considered in this qualitative, exploratory study, highlighting students' understanding of their own perfectionistic tendencies. Findings illustrate the manifestation of perfectionism as a double-edged orientation that supported persistence, quality, and achievement while also contributing to delay, exhaustion, health neglect, and compromised work-life balance. Moreover, findings of the study caution against framing perfectionism solely as an individual problem to be managed by students, as perfectionistic tendencies were also shaped by supervisory expectations, institutional norms, and broader socio-cultural pressures. Interventions should thus address both individual coping and the academic environments in which perfectionism is maintained.

## Supplementary Material

COREQ Checklist.docx COREQ Checklist.docx

Interview Schedule.docx Interview Schedule.docx

## Data Availability

Participants consented to the publication of de-identified study findings, including themes, subthemes, and selected anonymised quotations. However, consent was not obtained for the full interview transcripts or underlying qualitative dataset to be deposited in a publicly accessible repository. Given the sensitive and context-rich nature of the interviews and the potential risk of deductive identification, the supporting qualitative data are not publicly available.
