# Supplementary material for: “It pushes me, but it also drains me”: A qualitative study of perfectionism and well-being among doctoral students in India
Source: Int J Qual Stud Health Well-being. 2026 Nov 11;21(1):2760664. doi: 10.1080/17482631.2026.2760664 (PMC13757905; doi:10.1080/17482631.2026.2760664)
Supplement: Supplementary file 2 [file ZQHW_A_2760664_SM1420.docx]

**Validated Semi-structured Interview Schedule**

*(Validated by seven experts: licensed clinical psychologists, academicians, and qualitative research professionals)*

“Thank you for participating in the study. I am interested in understanding how perfectionism is experienced by PhD scholars in India. Now I will be asking you some questions about it. Please feel free to ask me to repeat or explain if something is unclear to you. In case any question makes you feel uncomfortable, please feel free to tell me, you are welcome to skip it, and we will move on to the next.”

“Before we begin our interview, I would like to know:

Are you willing to participate in my study?”

Yes / No

“Audio recording of the interview will be needed for study purposes. These recordings will remain strictly confidential. Do you consent for the interview to be audio recorded?”

Yes / No

**Please note that item 1 is for rapport building, whereas items from 2 to 9 are the targeted questions.**

1. Can you tell me a little about your PhD programme and your area of interest?
   a. What made you pursue a PhD?
   b. How has your PhD journey been so far?
2. When you hear the term ‘perfectionism’, what does it mean to you?
   a. With regard to the form you had completed, can you tell me about any parts that resonated with you?
   b. Based on your responses to the form, how, if at all, do you relate perfectionism to your own experiences?
   c. Can you describe the kind of expectations that you have or have set for yourself, particularly regarding your PhD work?
   d. To what extent are your expectations for your work usually met? Can you tell me more about that?
   e. How do you feel when your expectations for yourself are met?
   f. How do you feel when your expectations for yourself are not met?
3. What factors, if any, influence your striving for perfection in your PhD work?
   a. What role, if any, do people around you play in your perfectionistic tendencies?
   b. At any point, have you thought that cultural factors may have played a role in your drive for perfection? If so, what are these factors?
   c. Does the academic environment in India play a role in your experience of perfectionism? If so, can you describe some instances in which you have experienced such influences?
   d. Do you think your gender plays any role in your striving for perfection? If so, in what ways?
   e. Are there any personal factors that may play a role in your striving for perfection? Can you elaborate?
   f. Are there any other factors that may play a role in your striving for perfection? Can you describe them?
4. In what ways, if any, does perfectionism manifest or express itself during different parts of your research process?
   a. Can you describe any instances where perfectionism has been helpful during your PhD journey?
   b. Have there been instances where perfectionism has been particularly hindering in your PhD journey? Can you describe them?
5. In what ways, if any, does perfectionism influence your overall well-being and work–life balance?
   a. Has perfectionism influenced your emotional well-being? If so, in what ways?
   b. Have you noticed any ways in which perfectionism may have influenced your physical health?
   c. Has perfectionism influenced your relationships with people in your life? If so, can you elaborate on any such experiences?
   d. Have people given you any feedback about your perfectionism? If so, what are your thoughts about such feedback?
6. How, if at all, do you manage or cope with the emotional and psychological challenges associated with perfectionism?
   a. Can you describe any instances in which you used coping strategies to manage such challenges?
   b. How helpful or unhelpful have you found these coping strategies? Can you tell me about what worked and what did not work as well?
7. What kinds of support, if any, are available to you in managing experiences or challenges associated with perfectionism?
   a. In what ways do these sources of support help you?
   b. What additional support, if any, do you think would be helpful?
8. What aspects of perfectionism do you think are not commonly discussed or understood?
   a. Are there any misconceptions about perfectionism that you have encountered? If so, can you tell me about them?
   b. How has your understanding of perfectionism evolved over time, particularly during your PhD journey?
   c. In light of what you know now about perfectionism, is there anything you might have done differently? If so, can you describe what you might have changed?
   d. Based on your experience, what suggestions or recommendations would you give to other scholars who may have similar experiences?
9. Is there anything else you would like to share or discuss regarding your experiences with perfectionism as a PhD scholar?
